# Efficient coding predicts synaptic conductance

**DOI:** 10.1007/s00422-026-01060-6

**Published:** 2026-08-31

**Authors:** James V. Stone

**Affiliations:** University of Sheffield, Sheffield, England

**Keywords:** Information theory, Efficient coding, Energy, Synapse, Shannon

## Abstract

Retinothalamic and thalamocortical synapses are efficient in the sense that each synapse conveys as many bits per Joule as possible, but efficiency falls rapidly if synaptic conductance deviates from its natural value Harris et al. (2015, 2019). However, the manner in which efficiency falls with conductance remains unexplained. Recently, Malkin et al. (2026) showed that synaptic noise is minimised given the available energy, consistent with a minimal energy boundary. Here, this boundary is expressed in terms of Shannon’s information theory Shannon and Weaver (1949), which yields a model that predicts the efficiency values observed in Harris et al. (2015) across a 120-fold change in synaptic conductance ($R^2= 0.769$, p<0.001). This model also predicts that, for a synapse at its natural conductance, each pre-synaptic spike provides an average of 3.58 bits to its post-synaptic neuron, which is consistent with physiological values. Crucially, given the biophysical constraints that, a) synaptic efficiency is maximised at the natural conductance, and, b) synaptic noise variance is minimised in accordance with the minimal energy boundary, the proposed model contains no free parameters, so it is predictive rather than descriptive. The results presented here are consistent with the general principle that CNS neurons maximise information efficiency (bits per Joule), rather than information rate (bits per second).

## Introduction

The efficient coding hypothesis, as originally formulated by Barlow (1959), was primarily concerned with minimising signal redundancy, which maximises information rate (bits per second), rather than information efficiency (bits per Joule). However, Shannon’s information theory Shannon and Weaver (1949) states that the energy cost per bit of information increases exponentially with information rate (bits per second), which means that CNS neurons are subject to severe energy constraints (e.g. Laughlin et al. 1998). Consequently, it seems likely that the default currency used by evolution to assess biological fitness depends on information efficiency, rather than information rate.

**Fig. 1:**
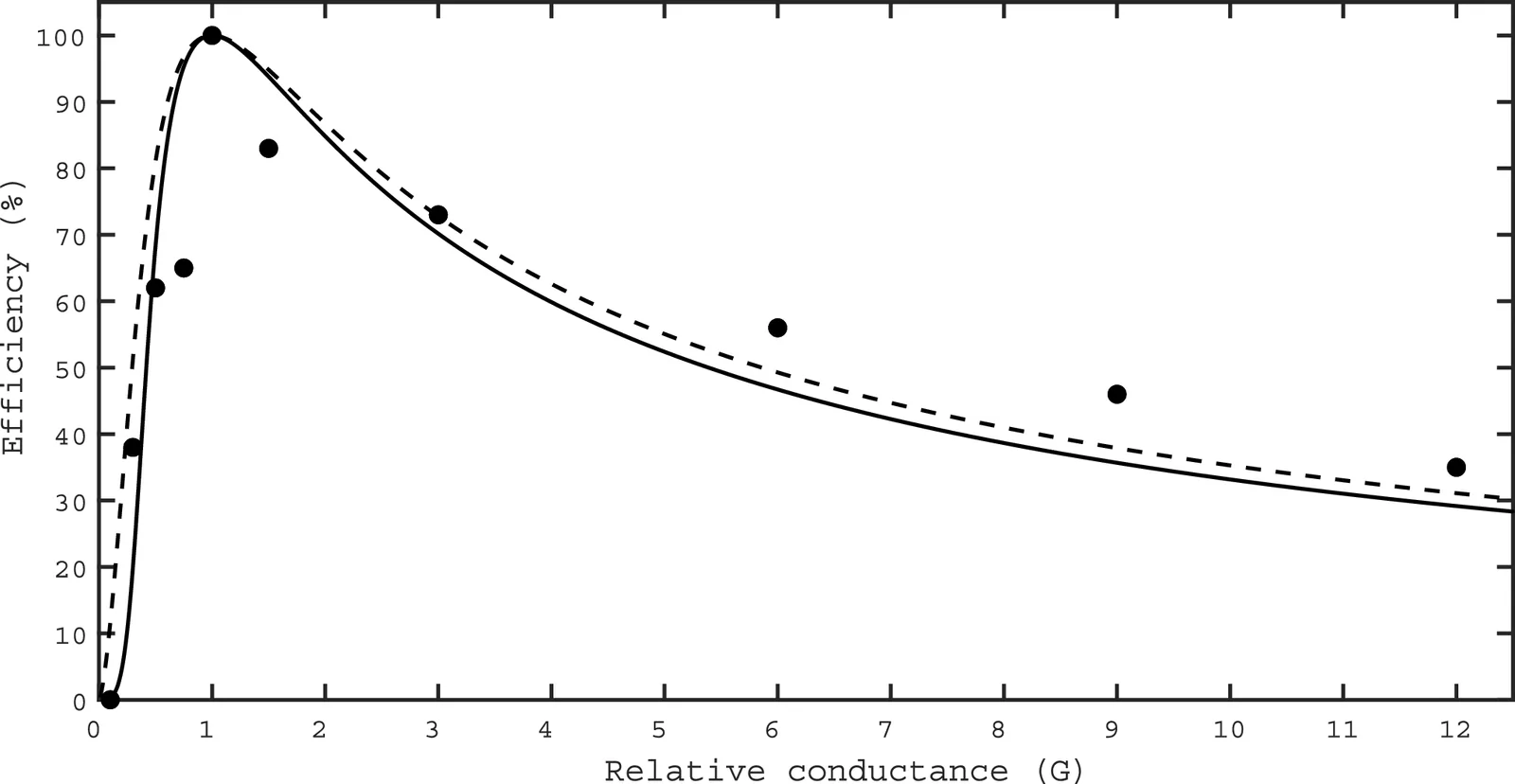
Maximum efficiency occurs at the natural synaptic conductance value, so G=1. The solid curve is ε (Eq. 22) with b=142.32. The dashed curve is $\varepsilon _{\text {Approx}}$ (Eq. 27), which assumes a high signal-to-noise ratio, with c=8.315. The black discs are data from Harris et al. (2015), where relative conductance values were reported as *G*=[0.1, 0.3, 0.5, 0.75, 1, 1.5, 3, 6, 9, 12]. The corresponding percentage efficiency values were measured from Figure 5E in Harris et al. (2015) as ε=[0.01, 38, 62, 65, 100, 83, 73, 56, 46, 35], re-scaled to percentage of the maximum observed efficiency (Eq. 22)

In support of this general principle, Harris et al. (2015, 2019) provide evidence for efficient coding in terms of bits per Joule. In their studies, the absolute conductances of synapses between, a) the optic nerve and the lateral geniculate nucleus, and, b) between lateral geniculate nucleus and spiny stellate cells in layer 4 of the primary visual cortex, were artificially altered from their natural values, and the information transmitted across the synapses was measured at each conductance value. It was found that, even though the information rate (bits per second) increases monotonically with increasing conductance, information efficiency (bits per Joule) is maximised at the natural conductance value, as shown in Fig. 1.

Recently, Malkin et al. (2026) demonstrated that neuronal data sets from five published studies conform to a minimal energy boundary condition, such that an energy budget determines an upper bound to synaptic noise precision. Their paper is summarised next.

Reliable transmission of information across a synapse requires that the variance in post-synaptic potentials is minimised. However, this variance ultimately depends on stochastic processes, such as the exocytosis of neurotransmitter vesicles from pre-synaptic release sites. Thus, energy is wasted if physiological parameters have values that do not minimise the variance in evoked post-synaptic potentials.

Three key physiological parameters are: 1) the number *n* of pre-synaptic active zones where neurotransmitter vesicles can be released, 2) the probability *p* that a vesicle will be released from any given site when a presynaptic spike arrives, and, 3) the size *q* of the electrical response produced in the post-synaptic neuron by a single vesicle. For an evoked post-synaptic potential $V_{EPSP}$, the mean evoked post-synaptic potential is

1 $$\begin{aligned} \mu= &  \textrm{E}[ V_{EPSP}] \end{aligned}$$

2 $$\begin{aligned}= &  npq. \end{aligned}$$

Assuming that neurotransmitter release behaves as *n* independent Bernoulli trials, with release probability *p* and quantal amplitude *q*, the noise variance across different evoked post-synaptic potentials is $\sigma ^2 = np(1-p)q^2$, which can be re-expressed in terms of the mean response μ

3 $$\begin{aligned} \sigma ^{2}= &  \text {var}(V_{EPSP}) \end{aligned}$$

4 $$\begin{aligned}= &  \mu (1-p)q. \end{aligned}$$

Precision is defined as the inverse of noise variance $\sigma ^{-2}$. Malkin et al. (2026) define a signal-to-noise ratio $\text {SNR}= \mu ^{2} / \sigma ^{2}$, and a minimal energy boundary, which predicts (in their Equation 20) that

5 $$\begin{aligned} \text {SNR}= &  \mu ^{2} / \sigma ^{2} \end{aligned}$$

6 $$\begin{aligned}= &  k E_{\text {Total}}^{5}, \end{aligned}$$

where *k* is constant for a given synapse. Therefore, the synaptic energy budget is

7 $$\begin{aligned} E_{\text {Total}}= &  \left( \frac{ {\mu ^2}}{ k\sigma ^2 } \right) ^{1/5} \text { Joules}. \end{aligned}$$

In essence, this synaptic energy budget comprises a dominant calcium pump–like cost and a smaller vesicle turnover–like cost. By analysing data from five studies, Malkin et al. (2026) found that synapses operate with energy budgets that lie just above the minimal energy boundary in Eq. 7.

## Predicting synaptic efficiency

A scatterplot of conductance *G* versus efficiency ε (measured as bits/Joule) from Harris et al. (2015) is shown in Fig. 1. In Harris et al. (2015), purely for convenience, efficiency values were fitted to a straight line for conductance values G<1, and fitted to an exponential function for conductance values G>1. Here, we express the minimal energy boundary proposed in Malkin et al. (2026) in terms of Shannon’s information theory Shannon and Weaver (1949) to derive a model that predicts with reasonable accuracy the efficiency values from Harris et al. (2015), plotted as data points in Fig. 1.

For a synapse with an energy budget of $E_{\text {Total}}$ Joules, which conveys *I* bits across a synapse, the absolute information efficiency is defined as

8 $$\begin{aligned} \varepsilon _{\text {Abs}}(G)\equiv &  {I} / { E_{\text {Total}}} \, \text { bits/Joule}. \end{aligned}$$

To derive the desired parametric function, mutual information *I* and energy $E_{\text {Total}}$ need to be re-expressed in terms of synaptic conductance.

First, Shannon’s equation for mutual information between channel inputs and outputs is re-expressed in terms of synaptic energy. Given that the input to a channel (synapse) has power *P* J/s, which is corrupted by noise with power *N* J/s, the mutual information between channel input and output is

9 $$\begin{aligned} I\le &  \log _{2}\left( 1 + P/N \right) ^{ {1}/{2}} \text {bits}, \end{aligned}$$

with equality if the channel is corrupted by additive white Gaussian noise η, which seems to apply to neural systems (Rieke et al. 1997) (using $\log _{2}$ ensures that information is measured in bits). If the channel input μ is constant then $P=\mu ^{2}$ J/s, and if the output is corrupted by Gaussian noise η with zero mean and variance $\sigma ^{2}$ then $N=\sigma ^{2}$ J/s. Thus, the signal-to-noise ratio (SNR) is effectively $P/N=\mu ^{2}/\sigma ^{2}$, so the mutual information between the channel input μ and output $\mu +\eta$ becomes

10 $$\begin{aligned} I= &  \log _{2}\left( 1 + \frac{\mu ^{2}}{\sigma ^{2}} \right) ^{ {1}/{2}} \text {bits}. \end{aligned}$$

This formulation of mutual information is usually associated with *signal detection theory* (Green and Swets 1966), which involves the probability of detecting a constant signal μ against background noise with variance $\sigma ^{2}$ (see Appendix).

If synapses operate at the minimal energy boundary then we can substitute Eq. 6 into Eq. 10, which yields

11 $$\begin{aligned} I= &  \log _{2}\left( 1 + k E_{\text {Total}}^{5} \right) ^{ {1}/{2} } \text {bits}. \end{aligned}$$

If the total root-mean-square (RMS) post-synaptic (signal plus noise) voltage is $V_{RMS}$ (measured across both active signalling events and periods when the pre-synaptic neuron is silent) and if the mean synaptic conductance is $G_{1} \, \Omega ^{-1}$ then Ohm’s law implies that the energy expended transmitting information across a synapse in Δt=1s is

12 $$\begin{aligned} E_{\text {Signal}}= &  V_{RMS}^{2}G_{1} \, \Delta t \text { Joules}, \end{aligned}$$

where $E_{\text {Signal}}$ is the energy demanded by the signal plus noise associated with a synapse.

In Harris et al. (2015), $G_{1}=\textrm{E}[g] \, \Omega ^{-1}$ is the mean conductance of a synapse, where this expectation was taken over 10 synapses, each of which was recorded for 125 s, and the relative synaptic conductance was defined as

13 $$\begin{aligned} G\equiv &  \frac{\text {artificially induced conductance}}{\text {natural conductance}} \,\, = \,\,\frac{G_{1}}{G_{0}}, \end{aligned}$$

so relative synaptic conductance *G* is measured in units of the synapse’s natural conductance $G_{0} \, \Omega ^{-1}$. Given that $G_{1} = G_{0} G \, \Omega ^{-1}$, Eq. 12 becomes

14 $$\begin{aligned} E_{\text {Signal}}= &  V_{RMS}^{2}G_{0} G \, \Delta t \end{aligned}$$

15 $$\begin{aligned}= &  {\beta G} \text { Joules}, \end{aligned}$$

where the energy constant is $\beta = V_{RMS}^{2}G_{0} \, \Delta t \text { Joules}$.

A critical assumption here is that the energy $E_{\text {Signal}}$ required to transmit information across a synapse is proportional to the energy budget

16 $$\begin{aligned} E_{\text {Total}}= &  {E_{\text {Signal}}} / {\lambda } \end{aligned}$$

17 $$\begin{aligned}= &  {\beta G} / {\lambda } \text { Joules}, \end{aligned}$$

where $\lambda =E_{\text {Signal}}/E_{\text {Total}}\le 1$. Therefore, the mutual information in Eq. 11 becomes

18 $$\begin{aligned} I= &  \log _{2}\left( 1 + k (\beta G/\lambda )^{5} \right) ^{ {1}/{2}} \text {bits}, \end{aligned}$$

where the signal-to-noise ratio is $\text {SNR}= k (\beta G/\lambda )^{5}$. For convenience, we represent the exponent 5 as the parameter γ,

19 $$\begin{aligned} I= &  \log _{2}\left( 1+ k (\beta G/\lambda )^{\gamma } \right) ^{ 1/2 } \end{aligned}$$

20 $$\begin{aligned}= &  \log _{2}\left( 1+ b G^{\gamma } \right) ^{ 1/2 } \text {bits}, \end{aligned}$$

where the constant $b=k (\beta /\lambda )^{\gamma }$ dictates the information capacity of a synapse at its natural conductance value (i.e. G=1).

Second, we express energy $E_{\text {Total}}$ in terms of relative conductance, which has been done in Eq. 17. Substituting Eqs. 17 and 20 in Eq. 8, defines the absolute information efficiency

21 $$\begin{aligned} \varepsilon _{\text {Abs}}= &  \frac{ \log _{2}\left( 1+ b G^{\gamma } \right) ^{ 1/2 } }{ \beta G / \lambda } \, \text { bits/Joule}. \end{aligned}$$

Thus, information capacity *I* (bits) increases logarithmically with conductance, whereas metabolic cost $E_{\text {Total}}= {\beta G} / {\lambda }$ increases linearly with conductance, which (taken together) predicts that absolute efficiency is higher in smaller synapses (see Discussion). In Harris et al. (2015), efficiency at the relative conductance *G* is defined as a percentage of the absolute efficiency at the natural conductance

22 $$\begin{aligned} \varepsilon\equiv &  \frac{\varepsilon _{\text {Abs}}(G) \times 100}{\varepsilon _{\text {Abs}}(G=1)} \,\,\, = \,\,\, \frac{\log _{2}\left( 1 + bG^\gamma \right) \times 100}{G \log _{2}(1 + b)}. \end{aligned}$$

It was found that setting G=1 in experiments maximised observed efficiency ε. To satisfy this constraint, we ensure that the maximum value of ε occurs at G=1. Equivalently, we set the value of *b* to ensure that dε/dG=0 at G=1. Differentiating ε with respect to *G*, setting the result to zero, and then setting G=1, yields

23 $$\begin{aligned} \frac{\gamma b}{1 + b}= &  \ln (1 + b). \end{aligned}$$

We then solve for *b* numerically, which yields a unique value for the constant b=142.32.

## Predicting synaptic efficiency: high SNR

If the signal-to-noise ratio is high then

24 $$\begin{aligned} \log _{2}(1+\text {SNR})^{1/2}\approx &  \log _{2}(1+\text {SNR}^{1/2}), \end{aligned}$$

and therefore the information in Eq. 20 is

25 $$\begin{aligned} I\approx &  \log _{2}\left( 1 + (b G^{\gamma })^{ 1/2 } \right) \end{aligned}$$

26 $$\begin{aligned}= &  \log _{2}\left( 1+ c G^{\alpha } \right) \text {bits}, \end{aligned}$$

where $\alpha =\gamma /2=2.5$, and the constant $c= k^{1/2} (\beta /\lambda )^{\alpha }$ dictates the information capacity of a synapse at its natural conductance value (i.e. G=1).

Substituting Eqs. 17 and 26 in Eq. 8 defines the absolute efficiency under the assumption of a high signal-to-noise ratio. Then, using the ratio of absolute efficiencies in Eq. 22, we obtain the approximate relative efficiency

27 $$\begin{aligned} \varepsilon _{\text {Approx}}= &  \frac{\log _{2}\left( 1 + c G^\alpha \right) }{G \log _{2}(1 + c)} \times 100, \end{aligned}$$

where the constant *c* is uniquely determined given that $d \varepsilon _{\text {Approx}}/ d G=0$ at G=1.

As in Eq. 23, setting the value of *c* to ensure $d{\varepsilon _{\text {Approx}}}/{d G}=0$ at G=1 yields

28 $$\begin{aligned} \frac{\alpha c}{1 + c}= &  \ln (1 + c). \end{aligned}$$

We then solve for *c* numerically, which yields a unique value for the constant c=8.315.

## Results

**Exact Model**. The exact model defined in Eq. 22 was evaluated using experimental data from Harris et al. (2015), as shown in Fig. 1. Using the information efficiency function ε (Eq. 22) with b=142.32 (which satisfies the constraint that $\varepsilon =100\%$ at G=1), and β=1, an *F*-test yields $R^2= 0.769$ with p=0.0004 (F(1,9)=29.91), even though no parameters were adjusted to fit the model to the data. This function is shown by the solid curve in Fig. 1.

**High SNR Model**. The model based on an assumption of high SNR (Eq. 27) was also evaluated using the same experimental data. Using Eq. 27 with c=8.315 (which satisfies the constraint that $\varepsilon _{\text {Approx}}=100\%$ at G=1), and β=1, a goodness-of-fit *F*-test yields $R^2= 0.724$ with p=0.0014 (F(1,9)=21.02). This function is shown by the dashed curve in Fig. 1.

Remarkably, both models predict the observed efficiency values reasonably well across a 120-fold change in relative synaptic conductance (G∈[0.1,12]). Note that the standard errors of estimated efficiencies increase when G>3 in Harris et al. (2015), which may account for the apparent loss of accuracy at higher values of *G*.

**Estimating Synaptic Information**. Given that synapses maximise efficiency by setting relative conductance to G=1 and that they operate at the minimal energy boundary, (so $G^{\gamma }=G^{5}=1$ and b=142.32, from Eq. 23), and therefore the information transmitted across a synapse (Eq. 20) is

29 $$\begin{aligned} I(G=1)= &  \log _{2}\left( 1+ b \right) ^{1/2} \end{aligned}$$

30 $$\begin{aligned}= &  \log _{2}\left( 1+ 142.32 \right) \end{aligned}$$

31 $$\begin{aligned}= &  3.58 \text {bits}. \end{aligned}$$

This suggests that each pre-synaptic spike that induces a post-synaptic voltage change provides an average of up to 3.58 bits to the post-synaptic neuron, which is strikingly similar to the information of 3.6±0.6 (mean ± standard deviation) bits/spike measured across retinal-LGN synapses in Reinagel and Reid (2000).

Note that 3.58 bits represents the average steady-state maximum information transfer of a maximally efficient synapse. In practice, synapses can transmit more (or less) than 3.58 bits. For example, if pre-synaptic spikes arrive in rapid succession then neurotransmitter vesicle release increases, which temporarily increases *G* above 1, so that I>3.58 bits.

## Discussion

Even though the minimal energy boundary demonstrates that synaptic noise variance $\sigma ^{2}$ is minimised for a given energy budget, it does not guarantee that information transfer *I* is maximised for a given energy budget; and therefore the minimal energy boundary does not guarantee that efficiency is maximised. Thus, from an information-theoretic perspective, the minimal energy boundary defines a necessary, but not sufficient, condition for maximising efficiency. For example, if some of the total energy $E_{\text {Total}}$ associated with a synapse is wasted then the signal amplitude μ could be reduced, which could reduce the mutual information *I*, so efficiency could be non-maximal, regardless of the noise variance $\sigma ^{2}$. Thus, whereas Malkin et al. (2026) showed that each synapse minimises noise variance, and Harris et al. (2015) showed that each synapse maximises efficiency at its natural conductance value, it has been shown here that each synapse maximises efficiency across a wide range of conductances.

Harris et al. (2015) report the mutual information between spike trains is 4.7 bits per spike, which implies a signal-to-noise ratio SNR ≈675. However, the smallest value of relative conductance $G=G_{1}/G_{0}=0.1$ used in Harris et al. (2015) implies a very low signal-to-noise ratio SNR=$b G^{\gamma }$=0.0014. Despite this, the approximate efficiency function $\varepsilon _{\text {Approx}}$ (Eq. 27), which is based on the assumption of a high signal-to-noise ratio, seems to predict observed efficiency values in the range G∈[0.1,1] reasonably well. This is because the efficiency value of approximately zero at G=0 is fixed by the laws of physics, and the efficiency value of 100% at G=1 is fixed by biophysical optimality; consequently, the $\varepsilon _{\text {Approx}}$ (and ε) is geometrically constrained about how it can vary between these two fixed efficiency values. In the intermediate range G∈[1,6], the function $\varepsilon _{\text {Approx}}$ continues to predict observed efficiency values reasonably well, which suggests that the synaptic signal-to-noise ratios are high, even at these intermediate relative conductance values. Finally, as relative conductance increases above G=6, the predictions of the approximation $\varepsilon _{\text {Approx}}$ become increasingly similar to those of ε, because $\varepsilon _{\text {Approx}}\rightarrow \varepsilon$ as $G \rightarrow \infty$.

As noted above, Eq. 21 predicts that efficiency is lower in large synapses. Given that size is proportional to conductance, this may explain the log normal distribution of synaptic sizes in the mammalian cortex (Bartol et al. 2015). This is consistent with deprivation studies which show that, under severe starvation, cortical neurons selectively reduce large (i.e. inefficient) synapses, rather than the more efficient small synapses (Padamsey et al. 2022).

### Limitations

The main result (Eq. 22) reported here is based on the minimal energy boundary Malkin et al. (2026) in Eq. 6. While it is difficult to test the validity of this boundary in general, it has been tested using five different data sets, which suggests it is fairly robust.

Despite the presence of a nonlinearity which triggers individual post-synaptic spikes, the post-synaptic firing rate is a monotonic function of the induced membrane potential across standard physiological regimes. This suggests that the spike train signal-to-noise ratios measured by Harris et al. (2015) are proportional to the corresponding signal-to-noise ratios between the pre-synaptic input and post-synaptic voltage, as assumed here.

The model proposed here is a reduced, steady-state abstraction rather than a dynamic model. This simplification is designed to capture the macroscopic, time-averaged constraints acting on a synapse over extended periods, which is consistent with the 125-second recordings analysed in Harris et al. (2015). While temporal dynamics and short-term plasticity alter instantaneous capacity, it is the metabolic costs of time-averaged quantities (e.g. conductance) that ultimately determine synaptic efficiency, and which are the main drivers of natural selection.

Levy and Baxter (2002) and Goldman (2004) showed that stochastic, low-probability neurotransmitter release transmission can increase efficiency by conserving pre-synaptic vesicle pools. However, multi-spike temporal analysis of short-term plasticity is beyond the scope of the steady-state structural model presented in this paper.

The proposed model is based on the assumption of uniform quantal amplitude, following Malkin et al. (2026). However, central synapses exhibit miniature EPSP coefficients of variation up to 0.5. As noted in the derivation of Equation 16 in Malkin et al. (2026), fluctuations in quantal amplitude variability could influence the value of the exponent γ (γ=5 here). Accordingly, the existence of non-uniform quantal variance should be taken into account in future research.

As stated above, a critical assumption is that the energy $E_{\text {Signal}}$ (Eq. 14) required to transmit information across a synapse is proportional to the energy budget $E_{\text {Total}}$ (Eq. 7). The fact that the efficiency function (Eq. 22) predicts the empirical data reasonably well in Fig. 1 provides support for the validity of this assumption.

## Conclusion

By expressing information efficiency in terms of synaptic conductance, the model proposed here predicts with reasonable accuracy the rate at which efficiency falls as synaptic conductance deviates from its natural value, subject to two boundary conditions:

1. efficiency is maximised at the natural conductance (G=1), as in Harris et al. (2015),
2. synaptic noise variance is minimised in accordance with the minimal energy boundary defined in Malkin et al. (2026).

The model also predicts that each pre-synaptic spike provides an average of 3.58 bits of information to its post-synaptic neuron, which is similar to the information of 3.6 bits/spike reported in Reinagel and Reid (2000).

The efficiency functions (Eqs. 22 and 27) plotted in Fig. 1 conform to the two boundary conditions above, so these functions contain no free parameters. Consequently, the plotted functions are predictive rather than descriptive, inasmuch as they have not been fitted to the data from Harris et al. (2015).

Overall, these results are consistent with the general principle that, while it is sometimes necessary for high capacity, but inefficient, peripheral sensory-motor neurons to maximise information rate (bits per second), evolution dictates that CNS neurons usually maximise information efficiency (bits per Joule) (Stone 2018).

## Data Availability

No datasets were generated or analysed during the current study.

## Appendix Shannon’s information theory and signal detection theory

Consider a noisy channel which is represented by a resistor of *R* Ω. The channel input signal is *X* volts (e.g. $X=V_{EPSP}$), and noise η volts is added to the signal as it passes through the channel, so the channel output is

1 $$\begin{aligned} Y= &  X + \eta \text { volts}. \end{aligned}$$

If the signal and noise are independent then the average output power is $\textrm{E}[Y^{2}]/R$ J/s. This can be expressed as the sum of the average input power, which is $P=\textrm{E}[X^{2}]/R$ J/s, plus the average noise power, which is $N=\textrm{E}[\eta ^{2}]/R$ J/s. For convenience, we assume R=1Ω, so that

2 $$\begin{aligned} \textrm{E}[Y^{2}]= &  \textrm{E}[X^{2}] + \textrm{E}[\eta ^{2}] \end{aligned}$$

3 $$\begin{aligned}= &  P+N \text { J/s}. \end{aligned}$$

We have now established that a channel with an input signal measured in volts implies signal power is measured in Joules per second (Watts).

In general, the mutual information between the channel input *X* and its output *Y* is the average output power $\textrm{E}[Y^{2}]$, expressed in units of the average noise power $N=\textrm{E}[\eta ^{2}]$

4 $$\begin{aligned} I\le &  0.5 \log _{2}\left( \frac{\textrm{E}[Y^{2}]}{\textrm{E}[\eta ^{2}]} \right) \text {bits}, \end{aligned}$$

with equality if the output has a Gaussian distribution, as is assumed here. Using Eq. 3, this can be written as

5 $$\begin{aligned} I= &  0.5 \log _{2}\left( \frac{ \textrm{E}[X^{2}] + \textrm{E}[\eta ^{2}] }{\textrm{E}[\eta ^{2}]} \right) \end{aligned}$$

6 $$\begin{aligned}= &  0.5 \log _{2}\left( 1 + \frac{\textrm{E}[X^{2}]}{\textrm{E}[\eta ^{2} ]} \right) \text {bits}, \end{aligned}$$

where the *average signal power* (input), expressed in units of the *average noise power*, is the *signal-to-noise ratio*

7 $$\begin{aligned} \text {SNR}= &  \frac{\textrm{E}[X^{2}]}{\textrm{E}[\eta ^{2}]} = \frac{P}{N}, \end{aligned}$$

which yields Shannon’s general definition of mutual information

8 $$\begin{aligned} I= &  0.5 \log _{2}\left( 1 + \text {SNR}\right) \text {bits}. \end{aligned}$$

The noise variance is

9 $$\begin{aligned} \sigma ^{2}= &  \textrm{E}[(\eta -\textrm{E}[\eta ])^{2}] \, = \, \textrm{E}[\eta ^2] - (\textrm{E}[\eta ])^2. \end{aligned}$$

It is generally assumed that noise has a mean of zero, so $(\textrm{E}[\eta ])^2=0$, so the average noise power is equal to its variance

10 $$\begin{aligned} {\textrm{E}[\eta ^{2}]}= &  \sigma ^{2}, \end{aligned}$$

so if the noise has a mean of zero then the signal-to-noise ratio becomes

11 $$\begin{aligned} \text {SNR}= &  \frac{\textrm{E}[X^{2}]}{ \sigma ^{2} }. \end{aligned}$$

Therefore, in general, the mutual information is

12 $$\begin{aligned} I= &  0.5 \log _{2}\left( 1 + \frac{\textrm{E}[X^{2}]}{ \sigma ^{2} } \right) \text {bits}. \end{aligned}$$

It will prove useful to note that the variance of the signal is

13 $$\begin{aligned} \sigma _{X}^2= &  \textrm{E}[(X-\textrm{E}[X])^{2}] \, = \, \textrm{E}[X^2] - (\textrm{E}[X])^2, \end{aligned}$$

so

14 $$\begin{aligned} \textrm{E}[X^2]= &  \sigma _{X}^2 + (\textrm{E}[X])^2, \end{aligned}$$

where the mean value of *X* is $\mu =\textrm{E}[X]$. Thus, the average signal power is the sum of the signal variance and the squared signal mean

15 $$\begin{aligned} \textrm{E}[X^2]= &  \sigma _{X}^2 + \mu ^2. \end{aligned}$$

Next, we consider two cases: 1) *X* is variable, which corresponds to Shannon’s approach, and is usually associated with *information theory*, and, 2) *X* is constant, which corresponds to Wiener’s approach, and is usually associated with *signal detection theory*.

### Shannon’s formulation: information theory

Using Shannon’s formulation, if *X* varies around a mean of $\mu =\textrm{E}[X]=0$ then, using Eq. 14, the average signal power equals it variance

16 $$\begin{aligned} \textrm{E}[X^2]= &  \sigma _{X}^2 - 0^2 \, = \, \sigma _{X}^2, \end{aligned}$$

and therefore Eq. 11 is

17 $$\begin{aligned} \text {SNR}= &  \frac{\sigma _{X}^{2}}{\sigma ^{2}}, \end{aligned}$$

so the mutual information in Eq. 12 can be written as Shannon’s classic formula

18 $$\begin{aligned} I= &  0.5 \log _{2}\left( 1 + \frac{\sigma _{X}^{2}}{\sigma ^{2}} \right) \text {bits}. \end{aligned}$$

### Wiener’s formulation: signal detection theory

Using Wiener’s formulation, if *X* has a constant value μ then its mean is also $\mu =\textrm{E}[X]$ and its variance $\sigma _{X}^2=0$. Therefore, using Eq. 14, the average signal power is

19 $$\begin{aligned} \textrm{E}[X^2]= &  0+ (\textrm{E}[X])^2 \, = \, \mu ^{2}. \end{aligned}$$

Therefore, the signal-to-noise ratio defined in Eq. 11 becomes

20 $$\begin{aligned} \text {SNR}= &  \frac{\mu ^{2}}{ \sigma ^{2} }, \end{aligned}$$

so the mutual information in Eq. 12 can be written as

21 $$\begin{aligned} I= &  0.5 \log _{2}\left( 1 + \frac{\mu ^{2} }{ \sigma ^{2} } \right) \text {bits}. \end{aligned}$$

In summary, given a noisy channel with input *X* and output Y=X+η (where the noise variance is $\text {var}(\eta )=\sigma ^{2}$), the mutual information between *X* and *Y* can be expressed either in terms of a varying channel input *X* (as $\textrm{E}[X^2] =\sigma _{X}^{2}$) or in terms of a constant channel input μ (as $\textrm{E}[X^2] =\mu ^{2}$).
